# Mitosis Inhibitors Induce Massive Accumulation of Phytoene in the Microalga *Dunaliella salina*

**DOI:** 10.3390/md19110595

**Published:** 2021-10-21

**Authors:** Yanan Xu, Patricia J. Harvey

**Affiliations:** Faculty of Engineering and Science, University of Greenwich, Central Avenue, Chatham Maritime, Kent ME4 4TB, UK; y.xu@greenwich.ac.uk

**Keywords:** phytoene, *Dunaliella salina*, mitosis inhibitors, herbicides, red light

## Abstract

Phytoene is a colourless natural carotenoid that absorbs UV light and provides antioxidant and anti-inflammatory activities as well as protection against photodamage. It is therefore valued for its skin health and aesthetic benefits by the cosmetic industry, as well as by the health food sector. The halotolerant green microalga *Dunaliella salina* is one of the richest sources of natural carotenoids. We have previously investigated the over-production of phytoene in *D. salina* after cultivation with the well-characterised mitosis inhibitor, chlorpropham. In this study, 15 herbicides with different modes of action were tested for their potential to promote phytoene accumulation. All herbicides showed different levels of capabilities to support phytoene over-production in *D. salina*. Most significantly, the two mitosis inhibitors tested in this study, propyzamide and chlorpropham, showed similar capacities to support the over-production of phytoene by *D. salina* cultures as phytoene desaturase inhibitors. The cellular content of phytoene increased by over 10-fold within 48 h of treatment with the mitosis inhibitors compared to untreated cultures. Results indicate a general effect of mitosis inhibitors on phytoene accumulation in *D. salina*. Furthermore, red light was found to significantly enhance the phytoene yield when used in combination with effective inhibitor treatments. Red light can be applied to maximize the production of phytoene from *D. salina*.

## 1. Introduction

Phytoene (C40 H64, (6*E*,10*E*,14*E*,16*E*,18*E*,22*E*,26*E*)-2,6,10,14,19,23,27,31-octamethyldotriaconta-2,6,10,14,16,18,22,26,30-nonaene) is a natural carotenoid and a rarity among naturally synthesized carotenoids, which are tetraterpenoids with a common C40 backbone of isoprenoid units. Phytoene is colourless and has a lower number (three) of conjugated double bonds compared to other coloured carotenoids that have at least ten conjugated double bonds [1]. Phytoene is the product of the first committed reaction of carotenogenesis, but a series of redox reactions normally converts it to carotenes rapidly [1]. Natural carotenoids have been reported to reduce the risks of cataracts, macular degeneration, neurodegeneration and some cancers because of their strong antioxidant capacity [2,3]. Like other carotenoids, phytoene has shown antioxidative effects that help defend against oxidative stress in humans and also show anti-inflammatory activity [4]. Furthermore, phytoene absorbs UV light maximally at 276, 286 and 297 nm in methanol/MTBE as solvent and can potentially protect against UV damage and diseases that are caused by harmful free radicals [5]. Phytoene is therefore valued in the cosmetic industry for its skin health and aesthetic benefits as well as in the health food industry. Moreover, phytoene shows higher bio-availability than other coloured carotenoids because it is released more efficiently from the food matrix, which is likely to occur because of its chemical structure and distribution in the food matrix [6].

**Figure 1:**
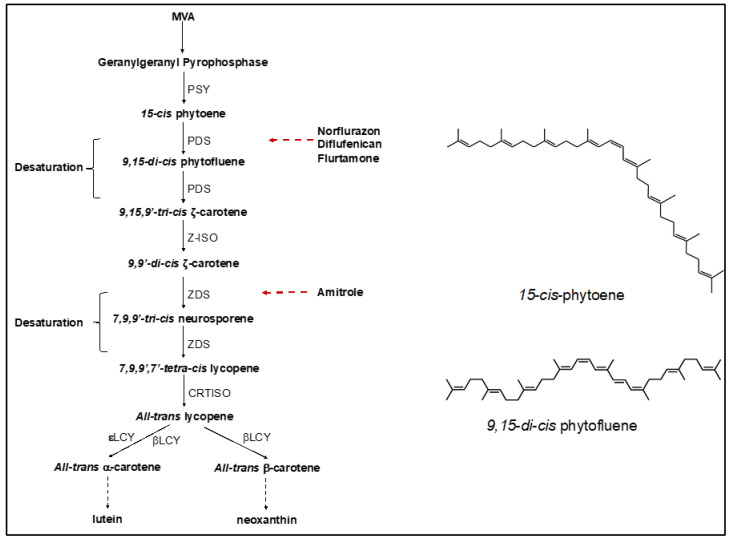
Carotenoid biosynthetic pathway showing key enzymes involved and specific sites of action by inhibitors.

In plants and algae, the first committed step in carotenoid biosynthesis is the chain-elongating condensation reaction of geranylgeranyl diphosphate (C20) to form 15-*cis* phytoene (C40), which is the first carotenoid product in the pathway [7]. This step is catalysed by phytoene synthase (PSY) and is considered a rate-limiting step of carotenogenesis [8]. The subsequent steps involve two desaturation reactions catalysed by phytoene desaturase (PDS), by ζ-carotene desaturase (ZDS) and the isomerization of the *cis* form to the *trans* form by carotenoid isomerase (CrtISO) to form all-*trans* lycopene [9]. All-*trans* lycopene is then cyclized into either β-carotene or α-carotene and catalysed by different groups of lycopene cyclases (LYC) [10]. The hydroxylation of β-carotene or α-carotene forms the xanthophyll products, which include zeaxanthin, antheraxanthin, violaxanthin and lutein, for further synthesis of their derivatives. The scheme in Figure 1 shows the carotenoid biosynthetic pathway and the key enzymes involved.

These reactions are ordered on membranes; an interruption of any one reaction leads to the accumulation of a metabolite from the corresponding precursor step and changes the carotenoid cellular composition. PDS is the most-used target for phytoene accumulation. It catalyses the oxidation of 15-*cis* phytoene to 9,15-di-*cis* phytofluene and 9,15,9′-tri-*cis*-z-carotene phytofluene in two sequential reactions which are coupled to the activity of a plastidial terminal oxidase (PTOX). These reactions use, respectively, plastoquinone as an intermediate electron acceptor and oxygen as a terminal electron acceptor. Consequently, PDS is redox-controlled and dependent on the redox state of the plastoquinone pool regulated by PTOX and oxygen. Plastoquinone mimics have been used successfully to induce the accumulation of phytoene in plant or algal cells; they bind competitively to the plastoquinone binding site of PDS, block phytoene desaturation and synthesis of 9,15-di-*cis* phytofluene and simultaneously reduce chloroplastic oxygen dissipation via PTOX [11,12,13,14].

The natural cellular levels of phytoene from different sources vary over a wide range. Among the most commonly known carotenoid-rich food sources, tomatoes contain the highest phytoene content (~5 mg/kg fresh weight) [5]. Phytoene products sourced from tomatoes have been commercialised in the cosmetic industry such as PhytoflORAL^®^ (Frutarom Industries Ltd., Herzliya, Israel). Algae are another potential source of the colourless carotenoids. The halotolerant marine microalga *Dunaliella salina* is one of the richest sources of natural carotenoids (up to 10% dry weight), the majority of which is β-carotene [15]. Under certain conditions, however, *D. salina* is able to produce significant amounts of phytoene. The over-production of phytoene has been achieved successfully in different algal species using the plastoquinone mimics norflurazon and fluridone, as well as the carboxamide diflufenican [16,17,18,19]. With the treatment of norflurazon, for example, *D. salina* accumulated up to 8% (*w*/*w*) of phytoene instead of β-carotene [17,20].

Although phytoene desaturase inhibitors used to be considered environmentally safe because they are used at very low concentrations and target an enzyme not found in animals [21], there have been increasing safety concerns: for example, norflurazon is difficult to mineralize or degrade and is a long-lasting contaminant in the environment [22,23].

Phytoene-accumulating *D. salina* has also been obtained by suppression of the PDS gene [24] and by deleting PDS genes or overexpressing the phytoene synthase genes, as has been reported in a bacteria strain *Deinococcus radiodurans* [25]. Over-expression of a phytoene synthase gene into the green microalgae *Chlamydomonas reinhardtii* results in over 2-fold increase of different carotenoids in the cells [26]. Taken together, these approaches focus on strategies that either up-regulate the upstream steps before phytoene synthesis or down-regulate the downstream steps after phytoene synthesis.

We have previously shown that a mitosis inhibitor, chlorpropham, which is widely used as a potato sprout inhibitor, will induce massive accumulation of phytoene in *D. salina.* Chlorpropham appears to disrupt the synchronised control between nuclear and chloroplast events in *D. salina*, which affects the recruitment of carotenogenic enzymes into biologically active, membrane-located metabolons [14]. The results suggest a new method with low toxicity for the production of colourless carotenoids by *D. salina*. We also demonstrated that red light promotes the production of carotenoids in *D. salina,* compared to white or blue light [12,14].

In the present study, apart from the commonly-known phytoene desaturase inhibitors and the proven mitosis inhibitor chlorpropham, 15 herbicides with different modes of action on functions in the metabolic pathway (either up-stream or down-stream of phytoene) were tested and the effects of red-light illumination on productivity were investigated.

The 15 herbicides were divided into six groups according to their modes of actions (Table 1). (i) Norflurazon, flurtamone and diflufenican are widely known PDS inhibitors known to induce phytoene accumulation. (ii) Amitrole, clomazone and cinmethylin are pigment inhibitors that are known to affect the synthesis of carotenoids but not function directly on PDS. Amitrole is a lycopene cyclase inhibitor that blocks the conversion of lycopene to β-carotene or α-carotene, but it has also been reported to inhibit the desaturation of ζ-carotene desaturase and cause the accumulation of ζ-carotene [19]. Clomazone inhibits deoxyxylulose 5-phosphate synthase and thus prevents the biosynthesis of isoprenoid needed to synthesize carotenoids. Cinmethylin inhibits 4-hydroxyphenyl-pyruvate-dioxygenase and leads to the blockage of the synthesis of plastoquinone, which is required by PDS in phytoene desaturation. (iii) Three cell division inhibitors, namely, propyzamide, dimethenamid and chlorpropham were tested since, as we previously demonstrated, the cell division inhibitor chlorpropham induced a massive accumulation of phytoene [14]. Propyzamide inhibits the polymerization of microtubules, while chlorpropham inhibits the organization of microtubules. Dimethenamid inhibits the synthesis of very long chain fatty acids. (iv) Auxins have been reported to regulate carotenoid biosynthesis in plants and algae [27,28]. In this study, aminopyralid is a synthetic auxin herbicide and binds at receptor sites that are used by natural growth hormones, which leads to the disruption of normal growth. Diflufenzopyr-sodium is an auxin transport inhibitor that affects the process of plant growth. (v) Metabolic inhibitors chlorsulfuron and glyphosate are two widely used amino acid synthesis inhibitors. They can cause a disruption of cellular homeostasis that enhances the generation of reactive oxygen species, which, in turn, affects carotenoid biosynthesis. Glyphosate has been previously shown to increase the lutein content in *D. salina* by more than 2-fold with a decrease in the β-carotene content [29]. (vi) Lastly, aminoethyl sulfate, a GABA transaminase inhibitor, and *cis*-1,2,3,6-tetrahydrophthalimide (CHDC), a germination inhibitor, both significantly affect cell development (which may further affect carotenoid biosynthesis) and were tested in this study.

## 2. Results

### 2.1. Phytoene Production with Combined Treatment of Different Inhibitors and Red Light

#### 2.1.1. Phytoene Desaturase Inhibitors

**Figure 2:**
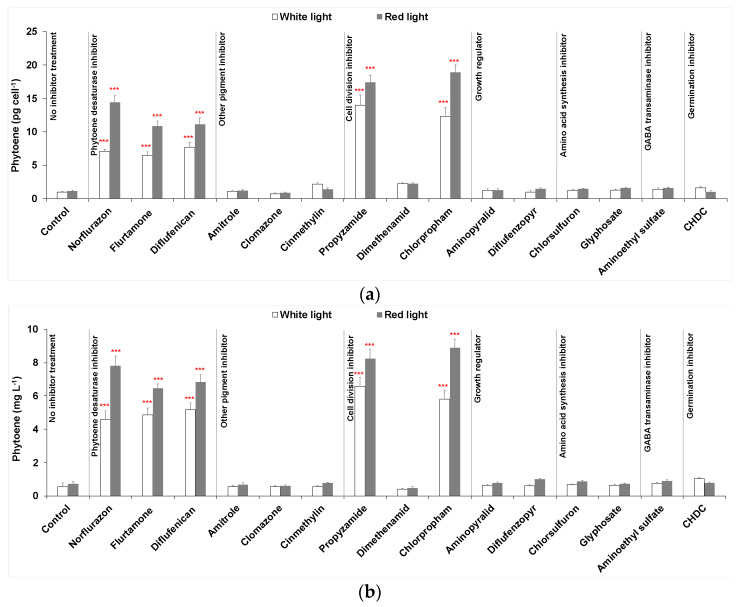

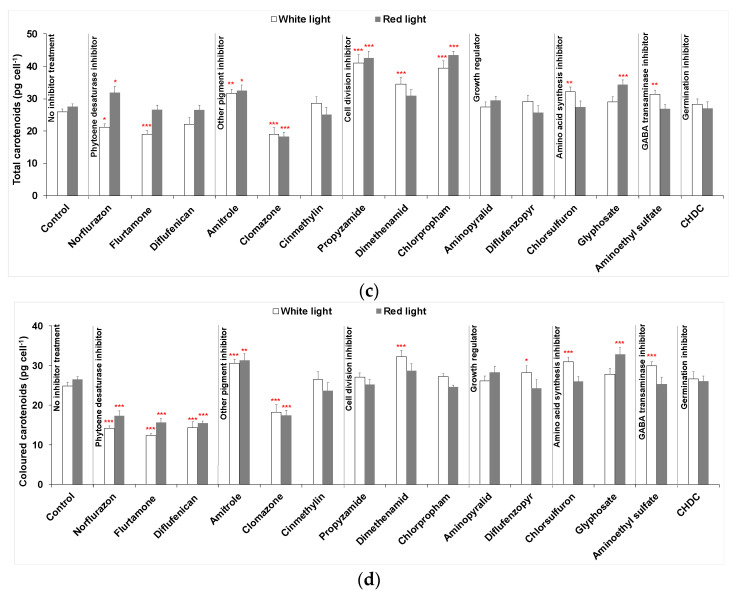
(**a**) Cellular content of phytoene, (**b**) phytoene yield, (**c**) cellular content of total carotenoids (sum of colourless and coloured carotenoids) and (**d**) cellular content of coloured carotenoids in *D. salina* cultures treated with different groups of herbicides: (i) phytoene desaturase inhibitors norflurazon, flurtamone and diflufenican at a working concentration of 5 µM; (ii) other pigment inhibitors of 50 µM amitrole, 20 µM clomazone and 20 µM cinmethylin; (iii) cell division inhibitors propyzamide, dimethenamid, chlorpropham at a working concentration of 20 µM; (iv) growth regulator inhibitors 250 µM aminopyralid or 20 µM diflufenzopyr-sodium; (v) amino acid synthesis inhibitors 100 µM chlorsulfuron or 50 µM glyphosate; (vi) 100 µM aminoethyl sulfate and 10 µM *cis*-1,2,3,6-tetrahydrophthalimide (CHDC). Optimal concentrations of each herbicide were pre-determined and concentrations with the highest phytoene yield were used. All cultures were illuminated either under white or red led light at 200 µmol m^−2^ s^−1^ for 48 h after adding herbicides. Results were analysed by one-way ANOVA in R with posterior Dunnett’s tests and compared to the control group. Asterisks represent different levels of significance (*** 0 < *p* ≤ 0.001, ** 0.001 < *p* ≤ 0.01, * 0.01 < *p* ≤ 0.05).

Three phytoene desaturase inhibitors (norflurazon, flurtamone and diflufenican) were used to treat *D. salina* cultures under either white LED light or red LED light, as is shown in Figure 2. After 48 h, the cellular content of phytoene and phytoene yield increased significantly for each treatment compared to control cultures maintained under white light without inhibitor treatment. The cellular content of phytoene was less than 1 pg cell^−1^ in control cultures but above 6 pg cell^−1^ in treated cultures under white light. The cellular content of total carotenoids in the treated cultures also increased compared to control cultures but was less significant than for the difference in phytoene content, as the increase in phytoene was accompanied by a decrease in the coloured carotenoids.

Under red light, significantly higher amounts of cellular phytoene content and total phytoene were produced than under white light for all three PDS inhibitors. Under white light, the levels of phytoene were similar for each inhibitor, but under red light, treatment with norflurazon gave a higher cellular content of phytoene (14.42 ± 0.95 pg cell^−1^) and phytoene yield (7.79 ± 0.58 mg L^−1^) than flurtamone or diflufenican.

#### 2.1.2. Pigment Inhibitors Other Than PDS Inhibitors

As shown in Figure 2, cultures treated by 50 µM amitrole or 20 µM clomazone did not show any increase in phytoene production, while the cellular content of phytoene in cinmethylin-treated cultures increased by ~2-fold compared to untreated cultures. However, the phytoene yield did not increase because cinmethylin also caused a significant reduction in cell growth. Red light increased phytoene production slightly in amitrole- or clomazone-treated cultures, while in cinmethylin-treated cultures, cellular phytoene content was lower under red light. Interestingly, red light cultivation supported a higher cell density than white light. Nevertheless, changes in phytoene as a result of treatment with these herbicides were marginal compared to changes caused by PDS inhibitors.

#### 2.1.3. Cell Division Inhibitors

All three herbicides were added to cultures to a final concentration of 20 µM. As shown in Figure 2, propyzamide and chlorpropham massively increased (≥10-fold) both the cellular content of phytoene and phytoene yield in the cultures and the increases were further enhanced by red light illumination. Both propyzamide and chlorpropham demonstrated even higher phytoene production capacities than the three PDS inhibitors. At the same time, total carotenoids also showed a more significant increase than the PDS treated cultures. On the other hand, dimethenamid increased the cellular content of phytoene by ~2-fold, but the phytoene yield decreased because of the loss of cell number. Red light did not improve either phytoene or total carotenoids in dimethenamid treated cultures.

#### 2.1.4. Growth Regulators

As shown in Figure 2, treatments with neither aminopyralid nor diflufenzopyr-sodium gave a significant increase in phytoene production or total carotenoids production.

#### 2.1.5. Amino Acid Synthesis Inhibitors

Treatments with 100 µM chlorsulfuron or 50 µM glyphosate caused a marginal increase in the cellular content of phytoene, total carotenoids and total chlorophylls marginally compared to PDS inhibitors (<1.5-fold increase). The phytoene content and total yield also increased slightly under red light illumination compared to white light.

#### 2.1.6. Other Inhibitors

Treatment of 100 µM aminoethyl sulfate increased both the cellular content of phytoene and the phytoene yield in cultures under either white light or red light. Treatment by 10 µM *cis*-1,2,3,6-tetrahydrophthalimide (CHDC), a germination inhibitor, increased cellular phytoene content (~1.5-fold) in *D. salina* cultures maintained under white light; however, no increase was shown in cultures under red light.

### 2.2. Cellular Composition of Carotenoids in Cultures Maintained under White or Red Light with Selected Heribicides

**Figure 3:**
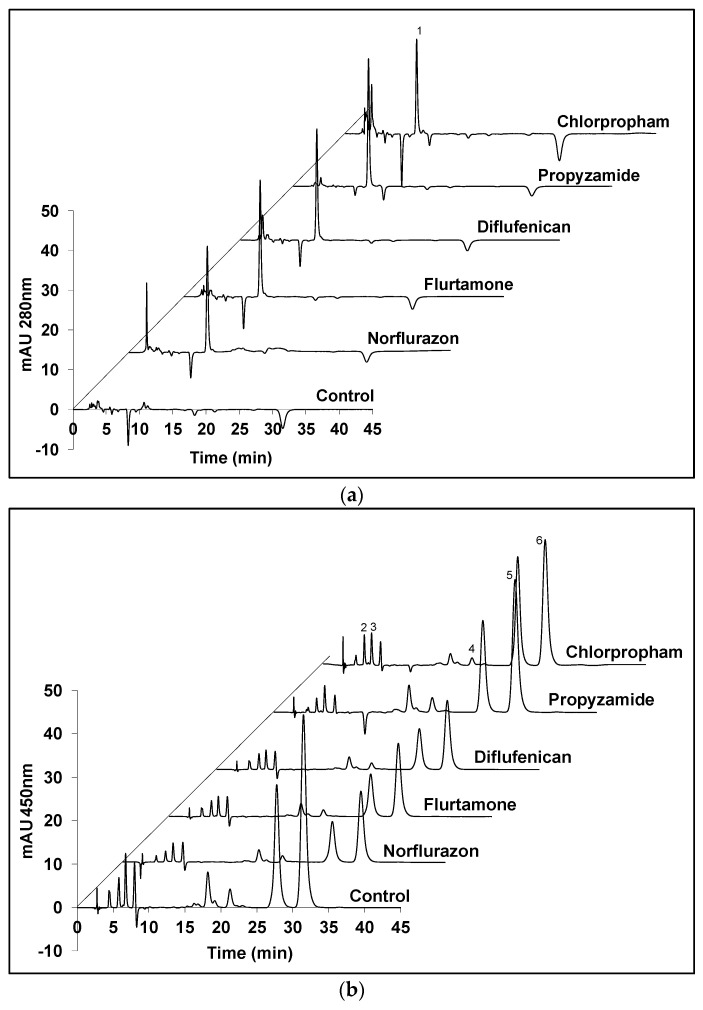
HPLC chromatograms of the carotenoids extracts from *D. salina* biomass grown under white light at 200 µmol m^−2^ s^−1^ and treated with herbicides that significantly increased phytoene production for 48 h. (**a**) Absorption at 280 nm detecting phytoene; (**b**) absorption at 450 nm detecting coloured carotenoids. Peaks: 1. phytoene, 2. lutein, 3. zeaxanthin, 4. α-carotene, 5. all-*trans* β-carotene, 6. 9-*cis* β-carotene.

Figure 3 shows HPLC chromatograms of carotenoid extracts from *D. salina* biomass treated with the five herbicides that significantly increased phytoene production (norflurazon, flurtamone, diflufenican, propyzamide and chlorpropham). The major carotenoids in the *D. salina* cultures, namely, phytoene, lutein, zeaxanthin, α-carotene and β-carotene, were quantified and their relative abundance was determined. As shown in Figure 3a, herbicide-treated cultures gave identical peaks corresponding to phytoene (peak 1) than in the control cultures. However, different levels corresponding to the coloured carotenoids were evident (Figure 3b). When compared to the control cultures, the three PDS inhibitors (norflurazon, flurtamone and diflufenican) showed a significant decrease in levels of the major coloured carotenoids, i.e., lutein (peak 2), zeaxanthin (peak 3), α-carotene (peak 4) and β-carotene isomers (peak 5 and peak 6), while propyzamide and chlorpropham did not show obvious changes in the content of coloured carotenoids.

**Figure 4:**
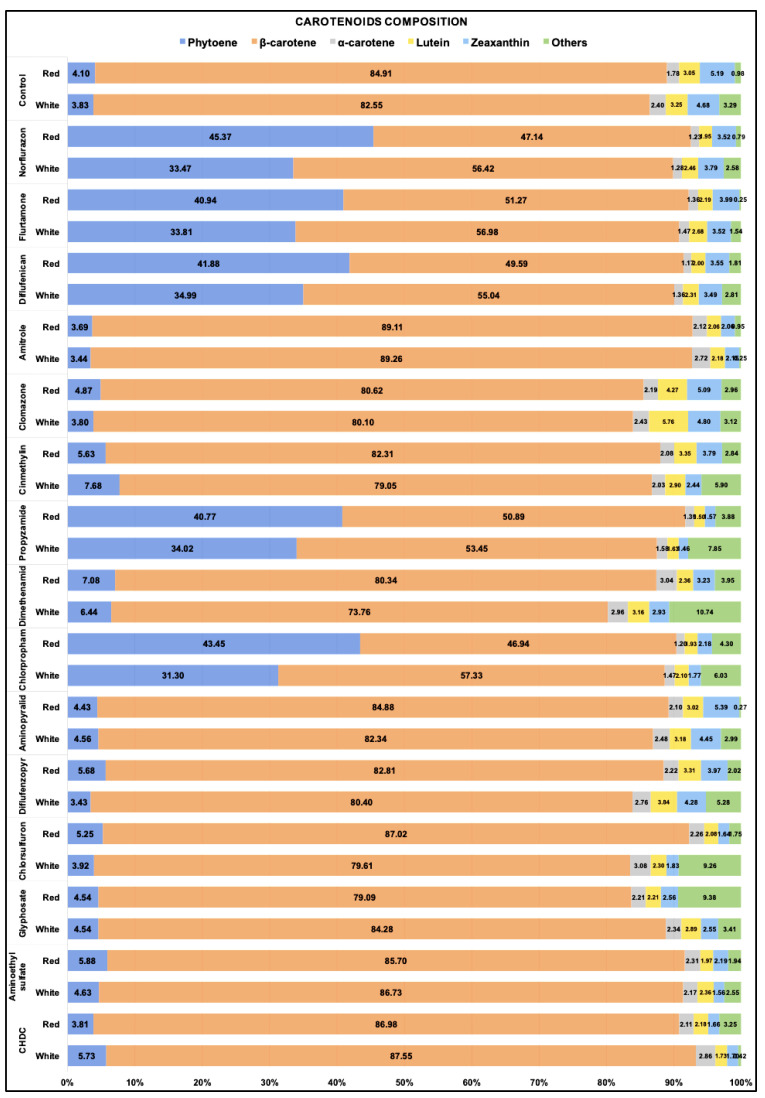
Relative carotenoids contents (% in total carotenoids) in *D. salina* cultures treated by different herbicides under white or red led light at 200 µmol m^−2^ s^−1^ for 48 h. Cultures of each condition were set up at least in triplets. Data are calculated by means of biological replicates (*n* ≥ 3).

The percentage of each carotenoid in total carotenoids was calculated for all the cultures treated with each of the 15 different herbicides and maintained either under white light or red light for 48 h. As shown in Figure 4, in control cultures without herbicide treatment, β-carotene (all-*trans* and 9-*cis*) was the major component and accounted for more than 80% of the total carotenoids, while the colourless phytoene amounted to less than 5% of the total carotenoids. Treatment with either norflurazon, flurtamone, diflufenican, propyzamide and chlorpropham increased the relative abundance of phytoene to above 30% under white light and above 40% under red light. Although some of the other herbicides, for example, cinmethylin and dimethenamid, also increased the phytoene composition, the increase was small.

## 3. Discussion

This study tested 15 different herbicides for phytoene production from *D. salina*, including three PDS inhibitors and a mitosis inhibitor chlorpropham already proven to induce massive accumulation of phytoene [14]. As expected, the three PDS inhibitors significantly increased (>10-fold) the cellular content of phytoene and reduced coloured carotenoids in *D. salina* culture.

In addition to the well documented PDS inhibitors, the two cell division inhibitors, propyzamide and chlorpropham, which disrupt mitosis by inhibiting either microtubule polymerization or organization, also induced a massive accumulation of phytoene (>10-fold). The difference between the PDS inhibitors and these two cell division inhibitors is that the accumulation of phytoene induced by the PDS inhibitors was accompanied by a corresponding decrease in the cellular content of other carotenoids, particularly β-carotene, in contrast to propyzamide and chlorpropham, which did not significantly change the cellular content of the coloured carotenoids. PDS inhibitors also block the formation of the colourless carotenoid phytofluene, which absorbs UV-A light, while propyzamide and chlorpropham led to the increase of phytofluene content. In our previous study, chlorpropham was shown to massively increase the phytoene and phytofluene content as well as the chlorophyll content [14]; the effect was not due to a direct binding inhibition of PDS or a stress response such as nutrient starvation but due to a breakdown in the carotenoid biosynthetic pathway that involves the division of the cell nucleus [14].

Both propyzamide and chlorpropham are widely used cell division herbicides. They have common properties in terms of function directed at microtubule spindle formation, which is needed for cell division. Both resulted in a similar over-production of phytoene in *D. salina*. Propyzamide belongs to the group of pronamide herbicides, which bind to tubulin and prevent the in vitro assembly of microtubules, causing the absence of microtubules in cells, while chlorpropham belongs to the group of carbamate herbicides, which disrupt the spindle microtubule organizing centres but usually do not cause the absence microtubules [30]. Another cell division inhibitor tested in this study, dimethenamid, which inhibits the synthesis of long chain fatty acids, did not show the same effect as propyzamide and chlorpropham. The results suggest that herbicides that have the same function as microtubule inhibitors have the potential to be used for the over-production of phytoene. Examples of other herbicides that inhibit polymerization of microtubules include benzamide, benzoic acid, dinitroaniline, phosphoramidate and pyridine herbicides (WSSA Group 3); examples of other herbicides that inhibit organization of microtubules include carbetamide and propham (WSSA Group 23).

This study also showed that inhibitors with different targets for activity can be applied to manipulate the composition of individual carotenoid in *D. salina* cells. Since all tested herbicides altered the cellular carotenoids’ composition to a greater or lesser extent, appropriate selection will be significant in relation to downstream processing for any given relative composition of carotenoids. In another study, diphenylamine (DPA), an inhibitor of β-carotene C-4 oxygenase, and glufosinate ammonium, a glutamine synthase inhibitor in the nitrogen metabolic pathway, increased the composition of β-carotene in *D. bardawil*, while glyphosate and DCMU significantly increased the composition of lutein [29]. Phytoene desaturase inhibitors bind to the same binding site as the plastoquinone, which acts as a cofactor for the function of phytoene desaturase [13]. Consequently, inhibitors, such as cinmethylin, that suppress the synthesis of plastoquinone would indirectly inhibit the activity of phytoene desaturase and are likely to induce the accumulation of phytoene with a decrease in other carotenoids. Moreover, since the activity of phytoene desaturase is controlled by the redox state of the plastidic plastoquinone pool [31], inhibitors that affect the redox state of the plastidic plastoquinone pool—that is, those affecting the activity of the photosynthetic electron transport in chloroplasts—are likely to regulate carotenoid synthesis, such as DBMIB.

The present study has shown, furthermore, that red light leads to further increases in the production of phytoene with herbicide-treated cultures of *D. salina*, which is in line with our previous study [12]. The enhancement is most significant when phytoene production is significantly increased by the three PDS inhibitors and the two microtubular inhibitors propyzamide and chlorpropham. Red light triggers the activity of phytoene synthase and hence enhances the production of carotenoids with the up-regulation of phytochrome-mediated synthesis of PSY transcripts [32]. Moreover, it has been shown that photoactivated phytochrome, when receiving light (preferably red light), leads to the degradation of PIF1 (phytochrome-interacting factor 1 that binds to the PSY promoter and represses PSY expression) [33], which indicates that red light would up-regulate PSY expression and increase carotenoids production. A combined use of appropriate herbicide treatment with red light illumination therefore offers an efficient way for the mass production of phytoene from *D. salina*.

## 4. Materials and Methods

### 4.1. Herbicides

A total of 15 herbicides were tested in this study. Norflurazon, diflufenican, flurtamone, amitrole, clomazone, cinmethylin, propyzamide, dimethenamid, chlorpropham, aminopyralid, diflufenzopyr-sodium, chlorsulfuron, glyphosate, aminoethyl sulfate, and *cis*-1,2,3,6-tetrahydrophthalimide were obtained from Sigma-Aldrich (Dorset, UK). Each herbicide was dissolved either in water or ethanol, based on its solubility, to make a stock solution of 1 m and then diluted to different working concentrations of 5–500 µM for each herbicide in the *D. salina* cultures. Optimised working concentrations with maximal phytoene production for each herbicide were screened and used for the treatment of algal cultures.

According to their modes of action, the herbicides tested are classified into six different groups: (i) phytoene desaturase inhibitors, (ii) other pigment inhibitors (rather than phytoene desaturase inhibitors), (iii) cell division inhibitors, (iv) growth regulators, (v) amino acid synthesis inhibitors and (vi) other inhibitors. The name, CAS number, site of action and the Weed Science Society of America (WSSA) group of each herbicide are listed in Table 1.

### 4.2. Algal Strain and Cultivation

*D. salina* strain CCAP 19/41 (PLY DF15) was obtained from the Marine Biological Association, UK (MBA). Algae were cultured in a Modified Johnsons Medium, as described by previous work [12]. *D. salina* cultures were grown to mid-log phase to a cell density of 0.2–0.5 million cells/mL and then different inhibitors were added. Treated cultures were maintained in an ALGEM environmental modelling labscale photobioreactor (Algenuity, Bedford, UK) at 25 °C. Cultures were treated with inhibitors for 48 h and illuminated with either white or red led light at 200 µmol m^−2^ s^−1^. The cell density of cultures was determined by counting the cell number of cultures using a haemocytometer after fixing the cells with 2% formalin. Biomass was collected regularly and analysed to determine the composition of cellular content of phytoene and other carotenoids.

### 4.3. Pigment Analysis

Phytoene and other carotenoids were analysed in cell extracts using a High-Performance Liquid Chromatography with Diode-Array Detection (HPLC-DAD), as described in previous work [12]. Commercial standards of individual carotenoids, including *all-trans* β-carotene, *all*-*trans* α-carotene, lutein, zeaxanthin and phytoene were obtained from Sigma-Aldrich, UK. Standards were dissolved in methanol or acetone to make a solution of 1 mg/mL and dissolved to a series of working solutions (5, 10, 25 and 50 µg/mL) to generate standard curves. To detect various pigments in the extracts, the DAD scanned wavelengths of 280 nm (colourless phytoene), 355 nm (colourless phytofluene), 450 nm (all coloured carotenoids including β-carotene, α-carotene, lutein and zeaxanthin) and 663 nm (chlorophylls). Total chlorophylls and coloured carotenoids were evaluated by extracting pigments from the harvested algal biomass of a 1 mL culture using 1 mL of 80% (*v*/*v*) acetone and absorbance was measured at 664, 647 and 480 nm, according to previous work [12].

### 4.4. Data Analysis

All cultures with each treatment were set up in triplicates and biomass analysis was carried out at least in triplicate (*n* ≥ 3). The collected data were calculated and analysed in R by one-way analysis of variance (ANOVA), compared to control cultures with no inhibitors. A *p* value < 0.05 was considered significant.

## 5. Conclusions

Fifteen herbicides that are known to modify carotenoid biosynthesis, but with different modes of action, were assessed for effects on the over-production of the colourless carotenoid phytoene in *D. salina*. Propyzamide and chlorpropham, two mitosis inhibitors that inhibit microtubule polymerization or organization, have the same capability to over-produce phytoene in *D. salina* as the phytoene desaturase inhibitors. It is most likely that mitosis inhibitors disrupt the synchronised control between cell nuclear and chloroplast events and the recruitment of carotenogenic enzymes into biologically active, membrane-located metabolons. Their use offers a novel method with low toxicity for the over-production of colourless phytoene. Furthermore, the use of herbicide treatments in combination with red light illumination further increased phytoene production, compared to white light illumination. Phytoene accumulation caused by mitosis inhibitors warrants further development.

## 6. Patents

This work is patented under WO/2019/097219 *Production of Dunaliella* [34].

## Figures and Tables

**Table 1:** A list of 15 herbicides tested in the study grouped by their modes of action according to the Weed Science Society of America (WSSA).

Herbicide	Herbicide Active Ingredient (IUPAC Name; CAS Number)	Site of Action	WSSA Group
	**(i) Phytoene desaturase inhibitors**		
**Norflurazon**	4-chloro-5-(methylamino)-2-[3-(trifluoromethyl)phenyl]pyridazin-3-one; 27314-13-2	Phytoene desaturase (PDS) inhibitor	12
**Diflufenican**	N-(2,4-difluorophenyl)-2-[3-(trifluoromethyl)phenoxy]pyridine-3-carboxamide; 83164-33-4		12
**Flurtamone**	5-(methylamino)-2-phenyl-4-[3-(trifluoromethyl)phenyl]furan-3-one; 96525-23-4		12
	**(ii) Other pigment inhibitors**		
**Amitrole**	1H-1,2,4-triazol-5-amine; 61-82-5	Lycopene cyclase inhibitor, ζ-carotene desaturase inhibitor	11
**Clomazone**	2-(2-chlorobenzyl)-4,4-dimethyl-1,2-oxazolidin-3-one; 81777-89-1	DOXP (1-deoxy-d-xyulose 5-phosphate synthase) inhibitor	13
**Cinmethylin**	1-methyl-2-[(2-methylphenyl)methoxy]-4-propan-2-yl-7-oxabicyclo[2,2.1]heptane; 87818-31-3	HPPD (4-hydroxyphenyl-pyruvate-dioxygenase) inhibitor (blockage of plastoquinone synthesis)	27
	**(iii) Cell division inhibitors**		
**Propyzamide**	3,5-dichloro-*N*-(1,1-dimethylprop-2-ynyl) benzamide; 23950-58-5	Microtubule polymerization inhibitor	3
**Dimethenamid**	2-Chloro-*N*-(2,4-dimethyl-3-thienyl)-*N*-(2-methoxy-1-methylethyl) acetamide; 87674-68-8	Long-chain fatty acid inhibitor	15
**Chlorpropham**	propan-2-yl *N*-(3-chlorophenyl) carbamate; 101-21-3	Microtubule organisation inhibitor	23
	**(iv) Growth regulators**		
**Aminopyralid**	4-amino-3,6-dichloropyridine-2-carboxylic acid; 150114-71-9	Synthetic auxins	4
**Diflufenzopyr-sodium**	sodium;2-[(*E*)-*N*-[(3,5-difluorophenyl)carbamoylamino]-C-methylcarbonimidoyl]pyridine-3-carboxylate; 109293-98-3	Auxin transport inhibitor	19
	**(v) Amino acid synthesis inhibitors**		
**Chlorsulfuron**	1-(2-chlorophenyl)sulfonyl-3-(4-methoxy-6-methyl-1,3,5-triazin-2-yl)urea; 64902-72-3	ALS (acetolactate synthase) inhibitors	2
**Glyphosate**	2-(phosphonomethylamino)acetic acid; 1071-83-6 sulfosate (glyphosate-trimesium) 2-(phosphonomethylamino)acetate;trimethylsulfanium; 81591-81-3	EPSP (5-enolpyruvyl-shikimate3-phosphate) synthase inhibitor	9
	**(vi) Other inhibitors**		
**Aminoethyl Sulfate**	2-Aminoethyl hydrogen sulfate; 926-39-6	GABA transaminase inhibitor (increased GABA levels)	-
***cis*** **-1,2,3,6-Tetrahydrophthalimide**	(3aR,7aS)-3a,4,7,7a-tetrahydroisoindole-1,3-dione, 1469-48-3	Germination inhibitor	-

## Data Availability

Data are contained within the article.
